# What underlies the observed hospital volume-outcome relationship?

**DOI:** 10.1186/s12913-021-07449-2

**Published:** 2022-01-14

**Authors:** Marius Huguet, Xavier Joutard, Isabelle Ray-Coquard, Lionel Perrier

**Affiliations:** 1 MINES Saint-Ètienne, Centre for Biomedical and Healthcare Engineering, 158 cours Fauriel, 42023 Saint-Ètienne, cedex 2, France; 2 Human and Social Sciences Department, Léon Bérard Centre, F-69008 Lyon, France; 3 Aix-Marseille Univ, CNRS, LEST, Aix-en-Provence, France; 4 OFCE, Sciences Po, Paris, France; 5 Univ Lyon, Leon Berard Cancer Centre, EA7425 HESPER, F-69008 Lyon, France; 6 Univ Lyon, Leon Berard Cancer Centre, GATE UMR 5824, F-69008 Lyon, France

**Keywords:** Volume-outcome causal effect, Epithelial ovarian cancer, Instrumental variable, Learning effect, Centralization of care, C31, C36, I11, I18, L11

## Abstract

**Background:**

Studies of the hospital volume-outcome relationship have highlighted that a greater volume activity improves patient outcomes. While this finding has been known for years, most studies to date have failed to delve into what underlies this relationship.

**Objective:**

This study aimed to shed light on the basis of the hospital volume effect on patient outcomes by comparing treatment modalities for epithelial ovarian carcinoma patients.

**Data:**

An exhaustive dataset of 355 patients in first-line treatment for Epithelial Ovarian Carcinoma (EOC) in 2012 in three regions of France was used. These regions account for 15% of the metropolitan French population.

**Methods:**

In the presence of endogeneity induced by a reverse causality between hospital volume and patient outcomes, we used an instrumental variable approach. Hospital volume of activity was instrumented by the distance from patients’ homes to their hospital, the population density, and the median net income of patient municipalities.

**Results:**

Based on our parameter estimates, we found that the rate of complete tumor resection would increase by 15.5 percentage points with centralized care, and by 8.3 percentage points if treatment decisions were coordinated by high-volume centers compared to decentralized care.

**Conclusion:**

As volume alone is an imperfect correlate of quality, policy-makers need to know what volume is a proxy for in order to devise volume-based policies.

**Supplementary Information:**

The online version contains supplementary material available at 10.1186/s12913-021-07449-2.

## Background

The Volume-Outcome Relationship (VOR hereafter) in health economics has been the subject of extensive investigation. To date, most of the studies have found that higher volume hospitals have better outcomes (e.g., lower mortality rates, longer progression-free survival) [1–11]. However, an observed correlation between the hospital volume and patient outcomes does not necessarily imply a causal impact of volume on outcomes. Luft et al. have proposed two hypotheses to explain how volume could correlate with outcomes [12]. The “practice-makes-perfect” hypothesis states that physicians and hospitals with a greater number of patients develop better skills through a learning process, while the “selective-referral” hypothesis is based on the opposite notion, namely that physicians and hospitals that have better outcomes attract more patients. The correlation between hospital volume and outcomes is likely to be a combination of these two hypotheses, making hospital volume endogenous in an outcome model. Furthermore, failing to properly control for differences in case-mix according to hospital volume of activities also makes hospital volume endogenous if they are correlated to patient outcomes. In the presence of endogenous hospital volume, instrumental variables allow estimation of a causal effect. To overcome these econometric issues, several studies have instrumented hospital volume of activities by the number of potential patients and other hospitals in a defined area [4, 6, 13, 14]. What most volume-outcome studies lack, however, is delving into what underlies the observed or estimated relationship. To the best of our knowledge, the existing literature has focused mainly on identification of the causal impact of volume on outcomes. Our contribution to the literature is to determine the extent to which the learning process implied by the “practice makes perfect” hypothesis could either relate to improvement in the clinicians’ skills at performing a specific procedure (e.g., a surgical intervention), or to a better ability of clinicians to choose the optimal treatment, especially for complex diseases with multiple treatment options.

We study the case of Epithelial Ovarian Carcinoma (EOC), which is characterized by a complex care pathway and a relatively low incidence rate (6.0 per 100,000 women in central Europe) with multiple treatment options that depend on the patient’s condition and the clinician’s decisions.

Although there has been extensive research on the VOR, few changes have been implemented in European countries regarding the organization of care (exceptions are a German pilot study [15], the centralization of ovarian cancer care in one health region in Norway [16, 17], the centralization of acute stroke patients in London [18], the centralization of acute hospitals in Denmark [17], and a volume-based policy to obtain authorization to perform several specific cancer surgeries in France [19]). For evaluating the VOR, we distinguish between a learning effect on the ability to perform a procedure and a learning effect on the ability to make the right decision. More specifically, we test whether there are differences in the use of neoadjuvant chemotherapy according to hospital volume of activities, and we examine whether they lead to a heterogeneous effect in regard to the complexity of the treatment received. Deciding between initial debulking surgery or neoadjuvant chemotherapy is a real challenge and there is no consensus regarding the correct decision-making process [20, 21]. Neoadjuvant chemotherapy is a treatment that is readily available for all hospitals and that does not involve expensive drugs. In this regard, the difference in the use of this treatment can be interpreted as a difference in the way clinicians decide the optimal treatment to be prescribed, and not as a difference in term of availability and access to the treatment for hospitals. To build volume-based policies, policymakers need to know what volume is a proxy for. Unraveling the process of learning and determining the extent to which the decisions by clinicians play a role in the volume-outcome relationship could have major implications and offer alternatives to centralized care for improvement of the overall quality of care.

The remaining part of this paper is structured as follows: section 2 describes the data and the empirical strategy; section 3 presents the results; section 4 provides a discussion of the results and limitations of this study and section 5 concludes.

## Materials and methods

### Data

Five French databases were used for this retrospective study. These comprised three clinical databases from clinical registries, the “Hospi Diag” public database of hospital characteristics, and open-access datasets from the National Institute for Statistics and Economic Studies (INSEE).

The clinical databases contained exhaustive datasets of patients in first-line treatment for EOC in 2012 in three regions of France (Calvados, Cote d’Or, and Rhone-Alps). These regions account for 15% of the metropolitan French population.

The databases include information on patient characteristics, such as age, cancer history (yes or no), patient residential postal codes, and — above of all — detailed information on the severity of the cancer: the presence of ascites, histology, the FIGO stage, and the tumor grade. The presence of ascites determines the level of liquid in the abdomen that can be identified at the time of diagnosis and that is likely to worsen the patient’s outcome. Epithelial ovarian tumors are classified into different histological subgroups based on several characteristics of the tumor [22]. Large differences in survival have been noted between different histological subgroups [23]. The FIGO stage relates to the size of the tumor, while the grade reflects the speed at which the tumor is growing.

**Fig. 1:**
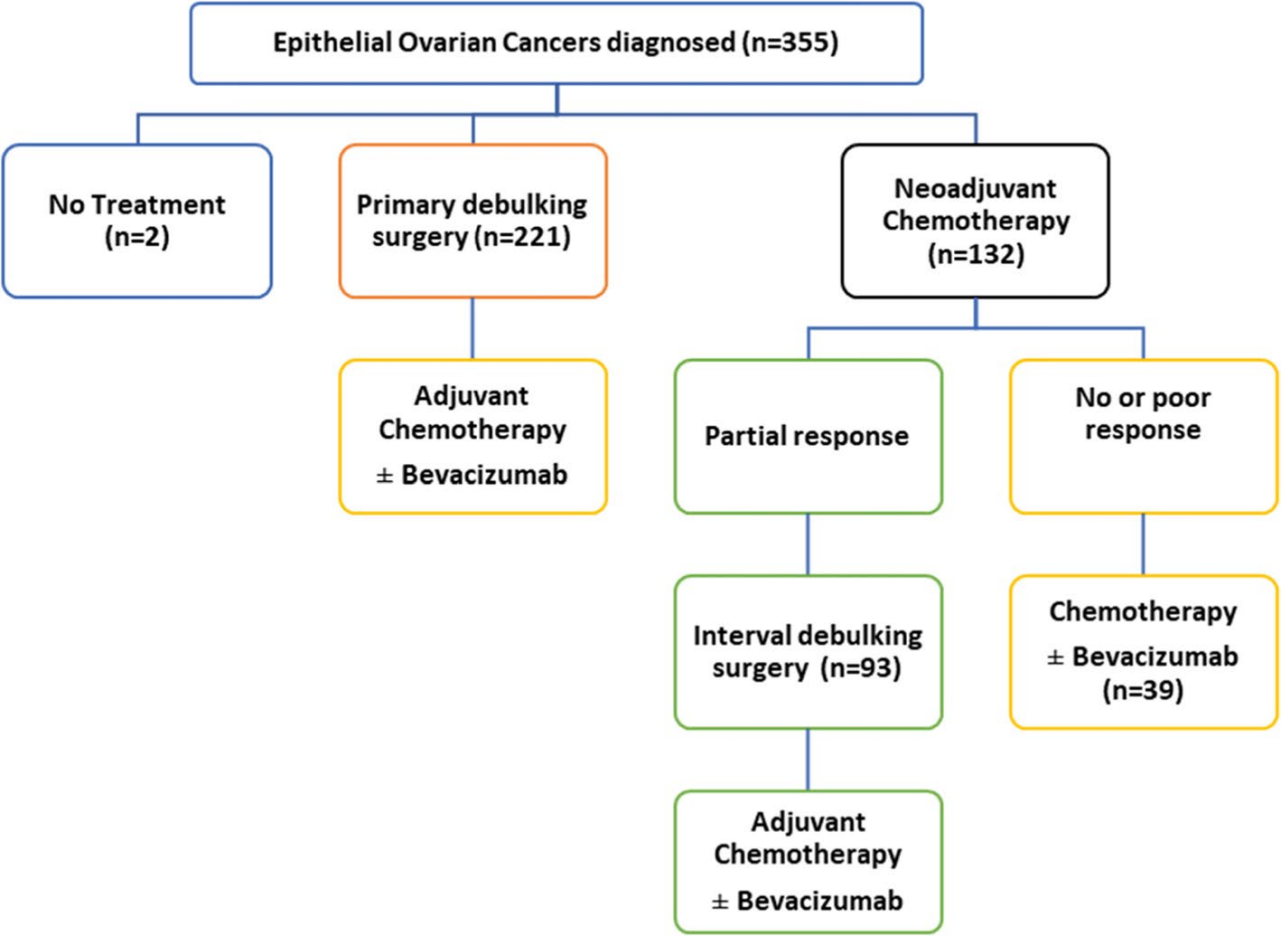
Care pathway of EOC patients

We obtained detailed information on first-line treatments for each patient. Figure 1 provides an overview of the treatment options for patients diagnosed with EOC. Primary surgery has been the standard treatment for decades. It aims to remove all of the tumor (i.e., complete tumor resection) without first performing chemotherapy. Neoadjuvant chemotherapy followed by surgery is a more recent treatment strategy for patients with advanced-stage EOC when they are found to have a low likelihood of complete tumor resection initially, and the goal of chemotherapy is to reduce the size of the tumor before the surgery in order to avoid a primary surgery that would be too aggressive for patients who are particularly ill [24]. Deciding between initial debulking surgery or neoadjuvant chemotherapy is a real challenge and is not consensual in the decision-making process [20].

Several hospital characteristics were taken into account, such as the share of the activity represented by oncology, the bed occupation rates in the surgery unit, the hospital’s accreditation by the National Authority for Health [25] and the Herfindahl-Hirschman Index (HHI). The hospital characteristics were only used for descriptive statistics, except the Herfindahl-Hirschman Index, which was included in the econometric specification.

In order to estimate the causal impact of hospital volume on patient outcomes, we also need to find instruments that correlate hospital volume while being independent of patient outcomes. We computed the distance between each patient’s residential postal code and the exact location of their hospital for first-line treatment. “Driving distances were computed using the function *mapdist* of the package *ggmap* in R statistical software.” We also included information about the municipalities, such as the median household income and the population density per square kilometer.

We used complete tumor resection as a quality indicator that is known to be the gold standard for first-line treatment [26]. For EOC patients, survival is strongly associated with the size of the residual disease after surgery [27]. As we only considered the hospital of first-line treatment in the data, complete tumor resection is the most direct outcome for comparing first-line treatments.^1^

### Econometric specification

The main objective of this study was to separate the positive impact of hospital volume on patient outcomes, and to differentiate between a learning effect on the ability to perform a procedure and a learning effect on the ability to make the right decision. We investigate whether there is heterogeneity of care pathways given the patient characteristics according to hospital volume of activities, in order to assess whether more experienced clinicians tended to have a different appreciation of the best treatment to prescribe for a specific patient. We concomitantly investigated how this link could contribute to the positive impact of hospital volume on patient outcomes.

As a benchmark, we first simply estimated the causal impact of hospital volume on our outcome of interest (i.e., complete tumor resection). To do this, one needs to take into account that hospital volume is very likely to be correlated with the error term in an outcome model, which would bias the estimated coefficients. Indeed, the endogeneity of hospital volume in the VOR model is mainly due to the incomplete observation of the patient’s state of illness: a part of the prognostic factors of EOC is likely omitted, as for the co-morbidities or for human breast cancer gene mutations (BRCA), which are known to increase the probability of developing ovarian cancer [28]. Other causes of endogeneity also warrant mention: the measurement errors related to the tumor staging - it has been shown that patients are more often properly staged at high-volume centers [29] - and the well-known simultaneous relationship between hospital volume and outcomes. In the presence of endogeneity that is induced by observed and/or unobserved factors, a method to estimate a causal effect is the instrumental variable. The idea is to find instruments variables that are strongly correlated to the endogenous variable, but that are strictly exogenous (i.e., uncorrelated with the error term). As it is commonplace in VOR studies, the hospital volume was instrumented by using a number of distance variables and we controlled for a set of patient characteristics that included age, a prior history of cancer, the presence of ascites, histology, the FIGO stage, and the tumor grade [4, 6, 13]. The full set of instruments were the logarithm of distance, an indicator for the closest hospital, the median net income in the patients’ municipalities, and the population density of the patients’ and the hospitals’ municipalities. See section 4.1 for a discussion of the reliability of our set of instruments. The results of this first model, designated as the “black-box model” are shown in additional Table 1 (Additional file 1). It can be seen that in this specification we could not identify a causal impact of hospital volume on patient outcomes when we do not take into account the heterogeneity in the care pathway.

**Table 1:** Hospital characteristics

	Top 10 High-Volume Hospitals	Low-Volume Hospitals (*n* = 64 hospitals)	*P*-value
Hospital volume of activity	15.80	3.08	0.000
Fraction of the hospital activity represented by oncology	38.42	11.40	0.000
Bed occupation rate in surgery (%)	81.40	80.90	0.983
Number of beds in surgery	373.67	115.62	0.001
Number of surgery rooms	37	11.63	0.001
Number of Surgeons	61.27	20.88	0.001
Number of Gynecologists and Obstetricians	18.16	7.10	0.005
Aggregate score for nosocomial infection prevention	87.25	85.14	0.476
Type of hospital (%):			
Private for profit	20	50	0.000
Private not for profit	10	6.45	
Public	0	38.70	
Teaching Hospital	70	4.85	
Accreditation (French National Authority for Health) (%):			
Accreditation	37.50	38.98	0.732
Accreditation with recommendations for improvement	37.50	22.03	
Accreditation with mandatory improvement	25	33.91	
Conditional accreditation due to reservations	0	5.08	

*Note*: The differences were analyzed using the Student’s t-test or the Chi-square test

The black-box model does not provide information about the process of learning that the relationship implies. In order to unravel this effect, we completed the original model by taking into account the care pathway decision and the care process. Thus, we now have several equations of interest (i.e., an outcome equation and several equations that describe the process of selection into different care pathway groups). To instrument hospital volume of activities in several equations, the typical approach would be to perform a 2SLS (i.e., two-stage least squares) estimation on each equation of interest. However, the power of such an estimation is limited by the available sample size. A natural way to model the endogenous hospital volume of activities in several equations (i.e., the care pathway and outcome) is to jointly link our equations of interest by allowing correlation between each error term [30]. To do this, one can assume a multivariate normal distribution of the error terms and estimate their covariance matrix by full-information maximum likelihood. However, for models with three or more equations, the cumulative normal densities of dimension three or higher must be computed [31]. We, in fact, used a more flexible approach that assumes that the error term in each equation includes a common random component in all of the equations and an independent idiosyncratic error term. The random component, which is assigned a parametric distribution, then has to be integrated into the likelihood function by Gaussian quadrature. Finally, we jointly estimated the following model using the procedure NLMIXED in SAS® (Statistical Analysis Software):

$$\left\{\begin{array}{c} Log\left({Volume}_i\right)={\beta}_1{X}_i+{\beta}_2{Z}_i+{\beta}_3{HHI}_i+{\gamma}_1{\alpha}_i+{\epsilon}_{1i}\\ {}{NACT}_i={\beta}_4{Volume}_i+{\beta}_5 Volume{2}_i+{\beta}_6{X}_i+{\beta}_7{HHI}_i+{\gamma}_2{\alpha}_i+{\epsilon}_{2i}\\ {} Log\left({TTS}_i\right)={\beta}_8{Volume}_i+{\beta}_9 Volume{2}_i+{\beta}_{10}{X}_i+{\beta}_{11}{HHI}_i+{\gamma}_3{\alpha}_i+{\epsilon}_{3i}\\ {}{Outcome}_i={\beta}_{12}{Volume}_i+{\beta}_{13}{\left( Volume\ \mathrm{x}\ \mathrm{NACT}\right)}_i+{\beta}_{14}{NACT}_i+{\beta}_{15}{X}_i+{\beta}_{16}{HHI}_i+{\gamma}_4{\alpha}_i+{\epsilon}_{4i}\end{array}\right.$$

Where *i* = 1, …, *N* are patient identifiers. *X*_*i*_ are the patients’ characteristics, including age, prior history of cancer, the presence of ascites, histology, the FIGO stage, and the tumor grade. *HHI*_*i*_ is the Herfindahl-Hirschmann index. The model is identified through our set of instruments *Z*_*i*_ for hospital volume, which are the same as for the black-box model. We suppose for the idiosyncratic error terms *ϵ*_1*i*_, *ϵ*_2*i*_, *ϵ*_4*i*_~*IIN* (0; 1) and *ϵ*_3*i*_~*Weibull* (λ; *k*). The individual’s random terms (i.e., *α*_*i*_), which is also assumed to be normally distributed, *α*_*i*_~*N* (0; 1) and independent of the idiosyncratic errors, represents the unobserved (to the econometrician) patient’s state of illness. This term, which links all of the equations together, provides the main source of endogeneity of the hospitals’ volume activities. *NACT*_*i*_ relates the first-line treatment prescribed for patients (i.e., neoadjuvant chemotherapy or primary surgery). For patients treated with neoadjuvant chemotherapy, *Log*(*TTS*_*i*_) is the time between the first cycles of chemotherapy until the surgery. To reduce the skewness of the hospital volume distribution, we employed a log-transformation of the hospital volume when it was used as a dependent variable. We also used a quadratic function of the hospital volume when it was used as an independent variable, to allow for a non-linear impact of the hospital volume on the dependent variable.

After estimation, this model is used for simultaneous prediction of the patient outcomes and the probabilities of being treated with neoadjuvant chemotherapy according to different scenarios of the organization of care.

As a robustness check, we also estimated our three equations of interest (i.e., NACT, Log(TTS), and Outcome) separately using a propensity score method, which is the other way of estimating a causal effect when selection is assumed to be only on observables factors. See Additional file 2 for the results and discussion of this alternative approach (additional table 2, additional table 3).

## Results

### Descriptive statistics

**Fig. 2:**
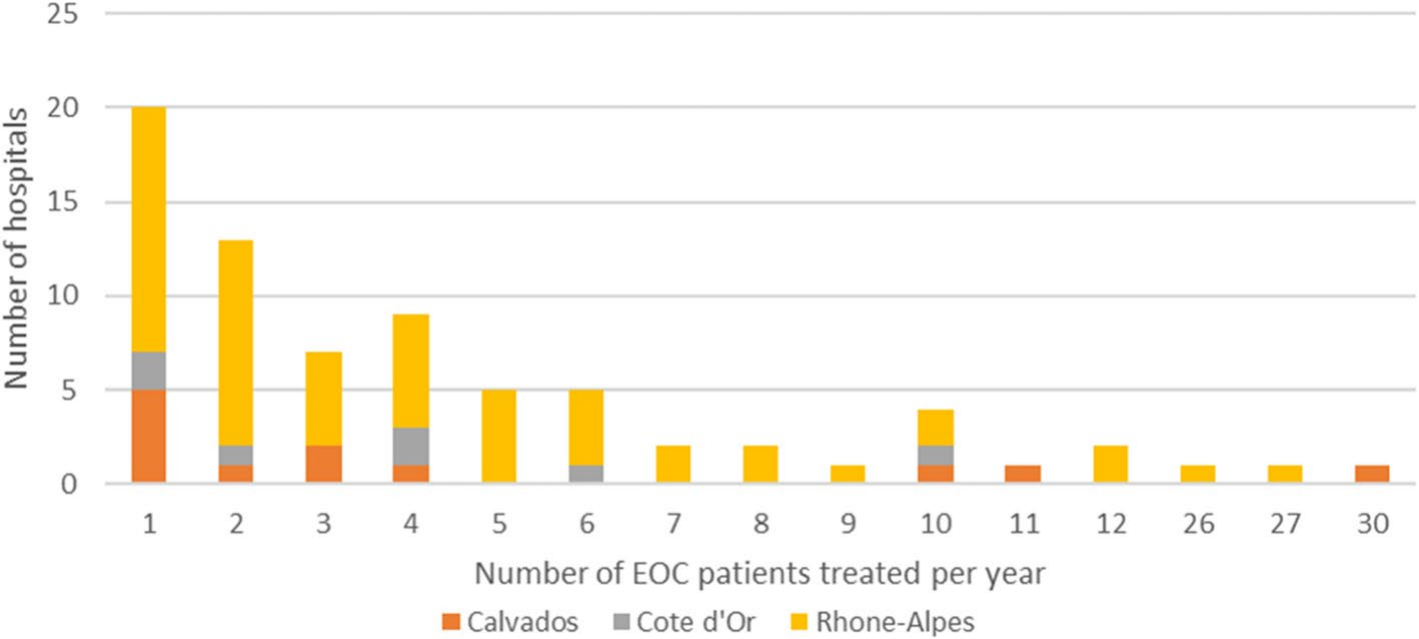
Distribution of hospital volume of activities

In 2012, 355 patients were identified in first-line treatment for EOC and they were treated in 74 different hospitals in the Calvados, Cote d’Or, and Rhone-Alpes region. The high number of hospitals compared to the low number of patients led to a mean hospital volume of activity of 4.8 patients treated in first-line per year and per hospital. A wide variation in the distribution is readily apparent in Fig. 2, which depicts the number of hospitals for each volume activity and by region.

Twenty of the 74 facilities (27%) had treated one patient in 2012, and 54 had treated five patients or less (73%). The top 10 hospitals with the highest volume activities treated 45% of the patients. An overview of the market structure and the geographical concentration of the providers is shown in additional Table 4 (Additional file 3). It can be seen that for about half of the patients there was at least one hospital within a radius of 10 km from their place of residence.

Table 1 displays the hospital characteristics according to their volume activity. In order to not make the descriptive statistics overly complex, we compared the 10 hospitals with the highest volume versus the other hospitals. It can be seen that the higher volume hospitals tended to be more specialized in oncology (*p* < 0.001), and they had a higher number of beds in the surgery unit (*p* < 0.001), a higher number of surgery rooms (*p* < 0.001), a higher number of surgeons (*p* < 0.001), and a higher number of gynecologists or obstetricians (*p* = 0.005). The type of hospital also appears to be a strong correlate of volume activity (*p* < 0.001), with 70% of the high-volume hospitals being teaching hospitals versus only 5% of the low-volume hospitals. Conversely, 50% of the low-volume hospitals were private for-profit hospitals, and 39% were public hospitals.

While the hospital characteristics differ according to hospital volume of activities, this is also the case for the patient characteristics (Additional file 4). Higher volume hospitals tended to treat the more severely ill patients and their patient intake was from a much larger area.

### Joint estimation of the full model

Table 2 displays the results of the full model, estimated jointly and integrated over the random-effects *α*_*i*_. Our set of instruments well impacts the choice of hospital according to the volume: patients treated at their nearest hospital were less likely to be treated in a high-volume hospital (*p* < 0.0001) and as expected, higher volume hospitals tended to receive patients from a larger area. The population density around hospitals also increased the likelihood of being treated in a high-volume hospital (*p* < 0.0001).

**Table 2:** Full model with individual random effect

	*Log (Volume)*	*NACT*	*Log (TTS)*	*Outcome*
Volume		0.1776^b^	−0.06186^a^	0.03918^a^
Volume^2^		−0.00398^b^	0.001565^a^	
NACT				1.3354^a^
Volume x NACT				−0.04495^b^
HHI	0.000069^a^	0.2656	−0.5855^a^	0.5549
Age	−0.00809^b^	0.03235^a^	0.002483	−0.01579^b^
Prior cancer	0.07331	0.4834^c^	−0.08445^c^	0.1469
Presence of ascites	0.04846	1.0399^a^	0.04917	−0.3440
Histology:				
HGSC	0.2772^b^	0.7841^a^	−0.04014	−0.02137
Other	Ref	Ref	Ref	Ref
Unknown	0.1161	1.3856^a^	−0.2636^a^	0.5823^c^
FIGO Stage				
I	Ref			Ref
II	0.1546			−0.1220
III	0.2014	Ref	Ref	−0.7611^a^
IV	0.3847^c^	0.4990	−0.06698	−1.6058^a^
Tumor Grade:				
1 or 2	Ref	Ref	Ref	Ref
3	0.08637	−0.03375	−0.08266	0.1141
Unknown	−0.2256	−0.1272	− 0.1305	−0.3597
Instruments:				
Closest	−0.5420^a^			
Log (Distance)	0.05269			
Population density	−0.00004^c^			
Density around hospital	0.000069^a^			
Median income	−0.00002			
Constant	2.0824^a^	−5.4872^a^	−4.1430^a^	0.9480^c^
Gamma	0.1882^c^	−0.8914^a^	0.3683^a^	0.02188
Log Likelihood	− 1377.1646			
AIC	2878.3			
Observations	294			

*Note*: High-Grade Serous Carcinoma (HGSC); Neoadjuvant Chemotherapy (NACT); Complete tumor resection (outcome); modality in reference (Ref); Herfindahl-Hirschman Index (HHI); Duration from the end of chemotherapy to surgery (TTS). Significant at 1, 5, and 10% is indicated as ^a^, ^b^, and ^c^, respectively

In the treatment equation (NACT), our variable of interest shows that patients treated in higher volume hospitals were more likely to be treated with neoadjuvant chemotherapy rather than primary surgery (*p* = 0.0125) with an inverted U-shaped effect (*p* = 0.0500). Although the effect of volume was positive, it declined per unit of volume as the volume increased. Furthermore, older patients, patients with ascites, HGSC, or an unknown histology compared to other histological subgroups were more likely to be treated with neoadjuvant chemotherapy rather than primary surgery.

In the duration equation (TTS), given a treatment with neoadjuvant chemotherapy, the time elapsed between the first chemotherapy and the surgery was shorter in higher volume hospitals (*p* < 0.0001), with a U-shaped effect (*p* < 0.0001). We also noticed that patients treated in hospitals with a higher HHI (i.e., less competitive areas) on average had a shorter time from the initiation of chemotherapy until surgery (*p* < 0.0001).

In the outcome equation (i.e., complete tumor resection), patients treated with neoadjuvant chemotherapy rather than primary surgery (*p* = 0.0004) were more likely to have no residual disease after surgery. Regarding our variables of interest, patients in primary surgery treated in higher volume hospitals were more likely to be fully debulked compared to patients who received the same treatment but in a lower volume hospital (*p* = 0.0014). While being treated in a higher volume hospital improved the outcome for patients in primary surgery, being treated with neoadjuvant chemotherapy reduced the difference in the likelihood of complete tumor resection according to hospital volume of activities (*p* = 0.0165). Other results: older patients and higher stage patients were less likely to be completely debulked after surgery.

### Predictions

To further illustrate the implications of the market structure on patient outcomes and on clinicians’ decisions, we simulated three scenarios reflecting different organization of care. After estimation, parameter estimates of the full model are used for simultaneous prediction of the patient outcomes and the probabilities of being treated with neoadjuvant chemotherapy according to different scenarios of the organization of care.

#### Scenario 1 - decentralized care

This scenario will be our reference point. It represents the ongoing organization of care whereby patients are treated at 74 different hospitals.

#### Scenario 2 - network formation

In this scenario, we predict an organization of care where first-line treatment decisions are discussed and coordinated by high-volume hospitals, but where the hospital of treatment does not change. As in the descriptive statistics, we used a threshold of 10 cases per year to define a high-volume hospital, which equates to comparing the ten hospitals with the highest volume to the other hospitals. We assume that the treatment decisions of patients in low-volume hospitals will be coordinated by the closest high-volume center to the patients’ residential municipalities.

#### Scenario 3 - centralization of care

In the third scenario, we assume that both the treatment decision and the treatment are performed at the nearest high-volume hospitals.

The results of the predictions based on our parameter estimates are displayed in Table 3. It can be seen that the rate of neoadjuvant chemotherapy among advanced-stage patients increased by 19.8 percentage points (pp) when the treatment decisions were made by high-volume centers. The rate of complete tumor resection among all patients would increase by 8.3 pp. if the patients were still treated in the hospital that they had chosen, and by 15.5 pp. if the care was centralized at high-volume centers.

**Table 3:** Results of the predictions based on parameter estimates of the full model

	Predicted patient outcome for all stages			Predicted first-line treatment for advanced stages disease		
	CC-1 or CC-2	CC-0	Rate of CC-0	PDS	NACT	Rate of NACT
Scenario 1: Decentralized	133	170	56.1%	125	72	36.5%
Scenario 2: Network formation	108	195	64.4%	86	111	56.3%
Scenario 3: Centralization	86	217	71.6%	86	111	56.3%

*Note*: Neoadjuvant Chemotherapy (NACT); Primary Debulking Surgery (PDS); Complete tumor resection (CC-0); Incomplete tumor resection (CC-1 or CC-2). First-line treatment is predicted only for advanced-stage patients, as primary surgery is the only treatment option for early stage

## Discussion

### Reliability of the instruments

To instrument the likelihood of a patient to being treated in a high-volume hospital, we used a function of the patient-hospital distance^2^ as our principal instrument. Distance has been widely used in the existing literature to instrument hospital volume of activities [1, 4, 6, 14]. The assumption here is that higher volume hospitals will receive patients from a much larger area compared to lower volume hospitals. We are confident of this assumption since higher-volume hospitals were more often not the closest hospital to the patient’s place of residence (Table 2). Higher volume settings are often located in or near big cities. To take into account that patients living in more populated areas will have greater access to these higher volume facilities, we included the population density of the patients’ municipalities as an instrument. Similarly, to take into account that hospitals located in more populated areas are more likely to have greater volume activities, we included the population density of the hospitals’ municipalities. There could also be inequalities in access to quality care for less wealthy patients who could not afford the expense incurred by a greater distance to the hospital. To take this into account, we included the median income at the municipality level. However, we could not identify a significant effect of median income, and we only found a weak association of hospital volume with the population density (*p* = 0.0872).

Based on the results presented in Table 2, we are confident of the reliability of our set of instruments since they appear to be good predictors of our endogenous variable (i.e., hospitals in more populated areas had greater hospital volume of activities (*p* < 0.001), as well as hospitals who caught patients in a broader area (*p* < 0.001). We are also confident of their validity since it is very unlikely that patients chose to live in a certain area according to the overall quality of the hospitals in that area. The added value of our database is that it includes detailed information about the severity of the disease, which reinforced the validity of the distance as instrument conditionally on these characteristics. It is not possible to perform a statistical test for the validity of the instrument in a non-linear model. We present some evidence that these instruments are likely to be valid in a linear model by estimating a linear probability model in a two-stage least square regression in order to perform a test of validity of the instrument. The Sargan (*p* = 0.3645) and the Basmann test (*p* = 0.3886) did not reject the null hypothesis according to which our instruments are uncorrelated with the outcome.

### Why do higher volume hospitals use neoadjuvant chemotherapy more often than primary surgery?

Unlike the black-box model, the joint estimation of the full model gives detailed information on the way patients were treated according to the volume activity of the hospital where they received treatment. We found that the higher volume hospitals were more likely to treat patients with neoadjuvant chemotherapy than by primary surgery (Table 2). Neoadjuvant chemotherapy is a treatment that is readily available for all hospitals that already have authorization to treat gynecological cancers (i.e., all of the hospitals included in this study), and it does not involve expensive drugs. In this setting, the difference in the use of this treatment can be interpreted as a difference in the way clinicians decide the optimal treatment to be prescribed, and not based on the availability and access to the treatment for hospitals.

We also identify differences in the time elapsed from the initiation of chemotherapy until surgery for patients who were treated with neoadjuvant chemotherapy (Table 2): higher volume hospitals tended to have a shorter duration (i.e., TTS). This result could have two distinct implications. It is possible that patients treated in higher volume hospitals received fewer cycles of chemotherapy on average, or alternatively be the result of shorter waiting times before surgery. Ultimately, both interpretations are likely to be related to the waiting times. The clinical guidelines for the number of cycles of neoadjuvant chemotherapy advocate that the use of 3 to 4 cycles is the appropriate way to treat advanced ovarian carcinoma [32]. For the patients considered in this study, the number of cycles varied from a minimum of 3 to a maximum of 10 cycles. The shorter duration underlined in our model should therefore not be interpreted as higher volume hospitals providing undertreatment. Thus, higher numbers of neoadjuvant cycles could also be related to waiting times and interpreted as a way to make patients wait for their surgery.

### Does the VOR only apply to patients treated with primary surgery?

While higher volume hospitals tended more often to use neoadjuvant chemotherapy rather than primary surgery, it appears that the difference in outcomes according to hospital volume of activities decreased for patients treated with neoadjuvant chemotherapy (Table 2). This could explain why we did not find that there was an impact of hospital volume on outcomes in the black-box model, where patients were pooled irrespective of the treatment that they received (Additional file 1). The joint estimation and the information on treatments allowed us to unravel this heterogeneous impact, while we would have concluded that volume and outcome are independent in the black-box model.

The heterogeneous impact of hospital volume according to the treatment received stems from a difference in the complexity of the procedure. The aim of neoadjuvant chemotherapy as first-line treatment is to avoid a surgical procedure that is too aggressive for the most severely ill patients. Thus, for this subgroup of patients, the use of neoadjuvant chemotherapy reduces the complexity of the surgery compared to a primary surgery. This reduction in the complexity of the surgical procedure could in part explain why we observed less or even no difference in outcomes according to hospitals volume activities for patients treated with neoadjuvant chemotherapy while we observed strong differences for patients in primary surgery. A remarkable result is that lower volume hospitals tended to benefit more from the use of neoadjuvant chemotherapy compared to higher volume hospitals, although they actually use it less. What is even more striking with this finding is that clinicians in higher volume hospitals are assumed to benefit from a learning effect due to the number of surgical procedures that they perform each year. They thereby develop greater skills and could hence be more able to perform a complex surgery compared to a less trained clinician at a lower volume hospital, although our data indicate that the clinicians in lower volume hospitals were, on average, more likely to perform complex surgery rather than use neoadjuvant to reduce its complexity.

### External validity

The main limitation of this study is the sample size, which was low due to the disease characteristics and due to the geographical area covered by this study, as well as the relatively old period of inclusion (i.e., patients diagnosed in 2012). It would be interesting to replicate this study with an exhaustive cohort of patients at the national level and to consider more recent data. Such databases are difficult to construct since detailed information on the severity of the disease is required in order to properly control for selection bias, which is usually not available in nationwide administrative data. Since we used an exhaustive cohort at a subnational level, we missed patients living in the area covered by this study but who had decided to be treated in a hospital that was not in the area covered by this study, and this could have potentially led to a sample selection issue. However, using administrative data from the Medical Information Systems Program (PMSI), we found that this sample selection bias was negligible in our cohort, since only 3.64% of the patients living in the Calvados, Côte d’Or, and the Rhone-Alpes regions in 2017 chose to be treated in a hospital that was not in the area covered by this study. To assess the external validity, we also checked the consistency of our data and results on patient characteristics with the existing literature. Globally, the results are in line with the existing literature, thus supporting the notion that the results of our study can be extrapolated to a certain degree. Indeed, we found that higher volume hospitals treated the more severely ill patients. This result is consistent with the existing literature on the VOR for EOC patients in the USA [33, 34]. We also found that the more severely ill patients and the patients treated in higher volume hospitals were more likely to be treated with neoadjuvant chemotherapy rather than primary surgery as first-line treatment. These results are consistent with a recent observational study on a cohort of 62,727 patients in the USA [35]. The distribution of hospital volume of activities we observed does not appear to be a specificity of the Calvados, Cote d’Or, or the Rhone-Alpes regions. Indeed, there was one hospital treating gynecologic cancers for every 111,638 residents in Calvados, one for every 154,845 residents in Cote d’Or, and one for every 113,174 residents in the Rhone-Alpes region in 2016 (source: National Institute of Statistical and Economic Information, French National Authority of Health). In comparison, there was one hospital treating gynecologic cancers for every 126,585 residents in the most populous region of France (i.e., Ile-de-France). We also lack information on socio-economic characteristics at the level of the individual, and instead relied on the income at the municipality level.

Based on the parameter estimates of the joint estimation of the full model, we have predicted several scenarios of the organization of care. These predictions aim to provide an insight into the variation in the outcomes and the care pathways that would arise if patients were reallocated in other hospitals based on their volume of patients. The first goal of this study is to unravel the process of learning implied by the volume-outcome relationship rather than build a model with a high predictive power. Thus, we assumed that patients will choose to be treated in their closest high-volume hospital. This assumption is conservative regarding the impact on patient access, but it should not undermine the variation in the quality of care and the care pathway highlighted in this study according to our three scenarios of organization of care. Finally, by using an instrumental variable approach, we estimate a local average treatment effect (LATE) for patients meeting our identification strategy. Thus, generalization of the results strongly depends on the reliability and validity of the instruments. As detailed in Additional file 2, a robustness check shows that results based on the propensity score approach are globally consistent with those from the joint estimation of the full model. In other words, the LATE estimate is close to the Average Treatment Effect on the Treated (ATT), which supports the reliability of the instruments being representative of hospital volume of activities in our population of interest.

### Policy implications

Centralized care at high-volume hospitals was the scenario that led to the highest average patient outcome (Table 3), and it has often been recommended in the literature [3, 8, 11, 16–18, 36, 37]. However, several barriers, such as the likely increase in patient travel distances, have prevented such a reform of the organization of care from being applied. Indeed, in our scenario, centralized care at the nearest high-volume center would increase the average distance traveled by patients from 39 km to 66 km. Moreover, centralized care at the nearest high-volume hospital requires that patients are no longer given the option of choosing their preferred provider. In health systems where patients have the option of choosing their hospital (e.g., France, the United Kingdom, and the United States), it has been shown that ignoring patient preferences when assessing the impact of such policies drastically underestimates the deterioration in patient access to care [38].

Another solution for volume-based policymaking could be to enhance cooperation between high- and low- volume providers. In this study, we showed that the expertise of high-volume providers in making treatment decisions plays an important role in the causal impact of hospital volume on patient outcomes for ovarian cancer care. Therefore, policymakers could incentivize clinicians in high-volume hospitals to cooperate and help clinicians in low-volume hospitals to make complex treatment decisions. With this alternative organization of care, patients would still be treated in their chosen hospital irrespective of whether it is a high-volume hospital. However, first-line treatment decisions for patients treated in low-volume hospitals would be discussed and coordinated by high-volume hospitals. This would have no impact on the distance traveled by patients, and it would also reduce inequalities in access to specialized care. Our findings support the notion that EOC patients would benefit from such an organization of care compared to the ongoing one. In terms of policymaking, it should be noted that in most cases this would require changing or adapting the payment scheme (e.g., such as activity-based payment) in order to allow for more cooperation between providers.

## Conclusion

This study provides evidence that clinicians’ decisions play an important role in the causal impact of hospital volume on patient outcomes for EOC patients. While the literature delving into what underlies this relationship is scarce, this is the first study to evaluate what proportion of the volume-outcome relationship may be induced by variations in clinicians’ decisions regarding which treatment path to follow. In terms of policymaking, this could have major implications, offering new possibilities to design volume-based policies, such as by cooperation between high- and low-volume providers for making treatment decisions. Note that centralization of care, whereby all patients would be treated in high-volume hospitals, is still the organization of care that leads to the greatest improvement in quality compared with the current organization (i.e., decentralized care) and to a scenario in which treatment decisions in low-volume hospitals could be coordinated by higher-volume providers. Nevertheless, the centralization of care also raises the issue of the inequalities in access to specialized care for patients.

More research needs to be undertaken before our findings can be extended to other diseases, especially regarding the organization of care for other complex diseases that could have some aspects in common with EOC. By contrast, for less complex diseases or when there is only a single treatment option, this type of organization of care would be less suitable.

## Supplementary Information

**Additional file 1.** Black-box model. Displays the results from the black-box model, which consists of a probit model with instrumented hospital volume, are indicative of the causal impact of hospital volume on outcomes.

**Additional file 2.** Propensity score analysis. Displays the results from a propensity score analysis, used as a robustness check. Use of a propensity score is indeed an alternative approach to an instrumental variable to estimate a causal effect in the presence of selection bias, which is based on different theoretical assumptions regarding the selection process.

**Additional file 3.** Share of patients that had at least ‘N’ hospitals treating gynecologic cancer to choose from in a radius of ‘K’ kilometers. Displays the share of patients that had at least ‘N’ hospitals treating gynecologic cancer to choose from in a radius of ‘K’ kilometers around the municipalities.

**Additional file 4.** Patient and municipality characteristics. Provides a comparison of patient and municipality characteristics between patients treated in low- and high-volume providers.

## Data Availability

The dataset analyzed during the current study is not publicly available due to the risk of the participants being identified, but are available from the corresponding author on reasonable request.

## Abbreviations

ATT: Average Treatment Effect on the Treated
EOC: Epithelial Ovarian Carcinoma
HGSC: High-Grade Serous Carcinoma
HHI: Herfindahl-Hirschman Index
INSEE: French National Institute for Statistics and Economic Studies
LATE: Local Average Treatment Effect
NACT: Neoadjuvant Chemotherapy
PDS: Primary Debulking Surgery
PP: Percentage Point
TTS: Time to surgery
VOR: Volume-Outcome Relationship
2SLS: Two-Stage Least Squares

## Footnotes

1 Use of survival could have introduced bias in the analysis, as some patients may have received secondary treatment at another hospital.

2 In this setting, we used the travel distance as an index of accessibility. Thus, it is a proxy for the true/unobserved travel time incurred by patients to get to their hospital. Naturally, the travel time could also be computed if one assumes a transportation mode. However, the decision of which transportation mode to assume is likely to strongly affect the travel time computed (i.e., especially in urban areas). Furthermore, for a given a transportation mode, there could also be substantial variation in the travel time according to the date and the time of travel (e.g., due to congestion).
