# Supplementary material for: What underlies the observed hospital volume-outcome relationship?
Source: BMC Health Serv Res. 2022 Jan 14;22:70. doi: 10.1186/s12913-021-07449-2 (PMC8760746; doi:10.1186/s12913-021-07449-2)
Supplement: Supplementary file 1 — Additional file 1. Black-box model. Displays the results from the black-box model, which consists of a probit model with instrumented hospital volume, are indicative of the causal impact of hospital volume on outcomes. [file 12913_2021_7449_MOESM1_ESM.docx]

**Additional File 1: Black-box model.**

In the table below, the results from the black-box model, which consists of a probit model with instrumented hospital volume, are indicative of the causal impact of hospital volume on outcomes. It can be seen that hospital volume and patient outcomes were independent when we controlled for the endogeneity of hospital volume if we do not take into account care pathways (p=0.224).

| Additional table 1: Black-box model | |
| --- | --- |
|  | Complete tumor  resection |
| Volume | 0.0234 |
| Age | -0.0096 |
| Prior cancer | 0.1623 |
| Presence of ascites | -0.2435 |
| Histology: |  |
| - HGSC | 0.1512 |
| - Other | Ref |
| - Unknown | 0.7475** |
| FIGO Stage: |  |
| - I | 1.3626*** |
| - II | 1.1339*** |
| - III | 0.8062*** |
| - IV | Ref |
| Tumor Grade: |  |
| -1 or 2 | Ref |
| - 3 | 0.0406 |
| - Unknown | -0.3511 |
| Intercept | -0.6483 |
| HHI | 0.5832 |
| Instruments | YES |
| Observations | 294 |
| Log Likelihood | -1212.33 |
| Note: High-Grade Serous Carcinoma (HGSC); modality in reference (Ref); Significant at 1%, 5%, and 10% is indicated as ***, **, and *, respectively. | |
