# Supplementary material for: What underlies the observed hospital volume-outcome relationship?
Source: BMC Health Serv Res. 2022 Jan 14;22:70. doi: 10.1186/s12913-021-07449-2 (PMC8760746; doi:10.1186/s12913-021-07449-2)
Supplement: Supplementary file 2 — Additional file 2. Propensity score analysis. Displays the results from a propensity score analysis, used as a robustness check. Use of a propensity score is indeed an alternative approach to an instrumental variable to estimate a causal effect in the presence of selection bias, which is based on different theoretical assumptions regarding the selection process. [file 12913_2021_7449_MOESM2_ESM.docx]

**Additional File 2: Propensity score analysis.**

As a robustness check, we employed a propensity score approach. Use of a propensity score is an alternative approach to an instrumental variable to estimate a causal effect in the presence of selection bias, which is based on different theoretical assumptions regarding the selection process. These methods rely on the assumption that the selection into the treatment (i.e., the patient being treated in a high-volume hospital in our case) is made only through observable characteristics. While instrumental variables allow for estimation of a Local Average Treatment Effect (LATE), propensity score methods allow for estimation of the Average Treatment Effect on the Treated (ATT). Among the several methods based on the propensity score, we used the Inverse Probability of Treatment Weighting (IPTW) method, which is more suited for small samples compared to matching methods since it does not reduce the sample size.

The IPTW method balances out the covariate of the two groups by weighting all of the patients in the database by the inverse of their propensity score. The propensity score is the conditional probability for a patient to be treated in a high-volume hospital, conditionally to observable characteristics. We determined this probability by fitting a logit model of an indicator variable denoting high- or low-volume hospitals on age, histology, FIGO stage, grade, cancer history, the presence of ascites, and the Herfindahl Hirschman Index (HHI). In order to define different groups and to fit the propensity score, we had to choose a threshold to define a high-volume hospital. Choosing a threshold is a constraining assumption, at least in the French health system, since no threshold has been officially defined. Thus, we used the same threshold as in Huguet et al. of 12 cases per year to define a high-volume hospital, which is the only study on the VOR for EOC patients in France [1]. We excluded predictive variables of outcomes that may depend on patient choice and subsequent interventions from this model, and we only controlled for patient characteristics at the time of diagnosis (i.e., prior to the patients receiving their first-line treatment). We used the stabilized weights for the estimation of the ATT, as proposed by Robins et al. [2].

Additional table 2 displays the balance in covariates between patients in high- and in low-volume hospitals after being weighted by the IPTW. It shows that there are no longer any differences between the two groups, thus indicating a good quality for the IPTW approach. Additional table 3 displays the results of the main equations of interest estimated separately and weighted by the IPTW. It can be seen that the results are fully consistent with those from the joint estimation of the full model, albeit with slightly larger standard errors (Table 2 in the main document). The increase in the standard errors does not impact the significance of the results, except for the impact of being treated in a high-volume hospital on Log(TTS) (p=0.355). The concordance of the results from the joint estimation with instrumented hospital volume activities and the IPTW (i.e., the LATE being close to the ATT) tends to support that the notion that our instruments are good predictors of the endogenous variable, and that the LATE estimate, to a certain extent, is representative of the impact on our population of interest.

| Additional table 2: Quality of the weighting by the IPTW | | | |
| --- | --- | --- | --- |
|  | High-volume hospitals | Low-Volume Hospitals | p-value |
| Age | 61.12 | 60.15 | 0.728 |
| Prior history of cancer (%) | 16.82 | 20.88 | 0.623 |
| Presence of ascites (%) | 71.03 | 71.02 | 0.999 |
| HHI | 0.2217 | 0.2233 | 0.938 |
| Histology (%): |  |  |  |
| - HGSC | 62.62 | 66.95 | 0.679 |
| - Other | 22.43 | 23.42 | 0.913 |
| - Unknown | 14.95 | 9.63 | 0.478 |
| FIGO Stage (%): |  |  |  |
| - I | 13.21 | 15.98 | 0.711 |
| - II | 6.61 | 7.75 | 0.835 |
| - III | 66.04 | 65.71 | 0.974 |
| - IV | 14.15 | 9.99 | 0.573 |
| Tumor Grade (%): |  |  |  |
| - 1 or 2 | 23.36 | 24.04 | 0.942 |
| - 3 | 71.03 | 74.82 | 0.698 |
| - Unknown | 5.61 | 1.14 | 0.330 |
| Note: High-Grade Serous Carcinoma (HGSC); Herfindahl Hirschman Index (HHI). The differences were analyzed using the Student’s t-test. | | | |

| Additional table 3: Results with inverse probability weighting | | | |
| --- | --- | --- | --- |
|  | NACT | Log(TTS) | Outcome |
| High-Volume Hospital (HVH) | 0.882*** | -0.075 | 1.388*** |
| HVH*NACT |  |  | -1.409** |
| NACT |  |  | 1.141** |
| Constant | -1.130*** | 4.849*** | -0.541** |
| Observations | 294 | 81 | 294 |
| Log Likelihood | -33.4 |  | -34.61 |
| Note: * p<0.1, ** p<0.05, *** p<0.01. The three models are estimated separately. NACT and Outcome are logistic regression, and Log(TTS) is an ordinary linear regression. | | | |

1. Huguet M, Perrier L, Bally O, et al. Being treated in higher volume hospitals leads to longer progression-free survival for epithelial ovarian carcinoma patients in the Rhone-Alpes region of France. *BMC Health Services Research*. 2018;18(3):1-11. doi:10.1186/s12913-017-2802-2

2. Robins JM, Hernán MÁ, Brumback B. Marginal structural models and causal inference in epidemiology. *Epidemiology*. 2000;11(5):550-560. doi:10.1097/00001648-200009000-00011
