# Supplementary material for: What underlies the observed hospital volume-outcome relationship?
Source: BMC Health Serv Res. 2022 Jan 14;22:70. doi: 10.1186/s12913-021-07449-2 (PMC8760746; doi:10.1186/s12913-021-07449-2)
Supplement: Supplementary file 3 — Additional file 3. Share of patients that had at least ‘N’ hospitals treating gynecologic cancer to choose from in a radius of ‘K’ kilometers. Displays the share of patients that had at least ‘N’ hospitals treating gynecologic cancer to choose from in a radius of ‘K’ kilometers around the municipalities. [file 12913_2021_7449_MOESM3_ESM.docx]

**Additional File 3: Share of patients that had at least ‘N’ hospitals treating gynecologic cancer to choose from in a radius of ‘K’ kilometers.**

The table below displays the share of patients that had at least *‘N’* hospitals treating gynecologic cancer to choose from in a radius of *‘K’* kilometers around the municipalities. It can be seen that 47% of the patients had at least one hospital within a radius of 10 kilometers from their place of residence. Approximately half of the patients had at least two providers that they could choose from within 20 kilometers of their place of residence.

| Additional table 4: The share of patients that have a choice of N hospitals located within K kilometers from where they reside. | | | | | |
| --- | --- | --- | --- | --- | --- |
| **Distance (K)**  **in Kilometers** | **Number (N) of hospitals** | | | | |
|  | **N=1** | **N=2** | **N=3** | **N=4** | **N=5** |
| **K=10** | 46.9 | 36.2 | 27.1 | 20.6 | 11 |
| **K=20** | 70.1 | 55.6 | 41 | 34.5 | 22.9 |
| **K=30** | 83.3 | 70.6 | 57.6 | 49.7 | 32.2 |
| **K=40** | 90.4 | 81.6 | 72 | 58.2 | 45.2 |
| **K=50** | 93.2 | 89.3 | 83.1 | 74.6 | 66.7 |
