# Supplementary material for: What underlies the observed hospital volume-outcome relationship?
Source: BMC Health Serv Res. 2022 Jan 14;22:70. doi: 10.1186/s12913-021-07449-2 (PMC8760746; doi:10.1186/s12913-021-07449-2)
Supplement: Supplementary file 4 — Additional file 4. Patient and municipality characteristics. Provides a comparison of patient and municipality characteristics between patients treated in low- and high-volume providers. [file 12913_2021_7449_MOESM4_ESM.docx]

**Additional File 4: Patient and municipality characteristics.**

| Additional table 5: Patient and municipality characteristics | | | |
| --- | --- | --- | --- |
|  | Top 10 High-Volume Hospitals (n=158 patients) | Low-Volume Hospitals (n=197 patients) | p-value |
| **Patient characteristics** |  |  |  |
| Age | 60.255 | 62.399 | 0.139 |
| Prior history of cancer (%) | 15.19 | 15.46 | 0.944 |
| Presence of ascites (%) | 67.72 | 58.25 | 0.068 |
| Primary inoperable (%) | 45.57 | 31.12 | 0.005 |
| Histology (%): |  |  |  |
| - HGSC | 55.70 | 44.67 | 0.013 |
| - Other | 23.42 | 38.06 |  |
| - Unknown | 20.88 | 17.27 |  |
| FIGO Stage (%): |  |  |  |
| - I | 17.99 | 30.09 | 0.080 |
| - II | 5.89 | 5.61 |  |
| - III | 60.64 | 52.55 |  |
| - IV | 15.48 | 11.75 |  |
| Tumor Grade (%): |  |  |  |
| - 1 | 6.96 | 17.77 | 0.007 |
| - 2 | 17.09 | 17.26 |  |
| - 3 | 61.39 | 46.70 |  |
| - Unknown | 14.56 | 18.27 |  |
| **Patient municipality** |  |  |  |
| Distance to hospital (km) | 42.92 | 36.21 | 0.414 |
| Hospital chosen is the closest (%) | 13.29 | 41.12 | 0.000 |
| European Deprivation Index | 3.21 | 2.82 | 0.414 |
| Population density | 1,477.50 | 981.62 | 0.047 |
| Median income | 20,653 | 20,593 | 0.857 |
| Note: High-Grade Serous Carcinoma (HGSC). The differences were analyzed using the Student’s t-test or the Chi-square test. | | | |
